# Cryotherapy for low rectal and anal cancer: recommendation and indications

**DOI:** 10.3389/fonc.2023.984145

**Published:** 2023-05-18

**Authors:** Xuejun Jiang, Zujin Ji, Xinyi Lei, Yingmei He, Fangjun Yuan

**Affiliations:** ^1^ Sinopharm Dongfeng General Hospital, Hubei University of Medicine, Shiyan, China; ^2^ College of Public Health, Hubei University of Medicine, Shiyan, China

**Keywords:** rectal cancer, cryoablation, organ preservation, indications, recommendation

## Abstract

Low rectal cancer is a common gastrointestinal malignancy. Organ preservation in the treatment of low rectal cancer is a challenge. By combining surgical resection with freezing—a complementary treatment for low rectal cancer—the anus can be preserved in some patients. However, we lack unified standards for colorectal cancer cryotherapy. Our hospital has been treating patients with cryotherapy since 1976. In our department, the indications for and contraindications to low rectal and anal cancer treatment are well established. In this paper, we summarize the indications for and contraindications to cryotherapy for colorectal cancer by reviewing the literature, drawing on our experience, and considering current imaging and histological techniques. Our aim is to facilitate clinical discussion and promote appropriate treatment.

## 1 Background

Colorectal cancer is a common disease. Its incidence is increasing year on year, and, according to the Global Cancer Report 2020 (1), it is the third most commonly diagnosed cancer and has the second highest mortality rate among all cancers. Low rectal cancer (i.e., any tumor lying <5 cm from the anal verge) is the most common type of rectal cancer (2), and it requires a complex treatment strategy based on surgery. Treatment, especially brachytherapy, adversely affects quality of life (3). Patients treated for low rectal cancer require a permanent stoma (4). Preservation of the anus and its function remains a key challenge in the treatment of low rectal cancer. Cryotherapy as a complementary treatment for low rectal cancer was introduced in the 1970s. Cryotherapy, which is based on the Joule–Thomson effect, is the ablation of tumors by low temperature. It has the advantages that it is easily administered and requires only local anesthesia or Sacral anesthesia, and the patient makes a rapid recovery, which go some way towards solving the above-mentioned problems. However, because of a lack of knowledge of the indications, adoption of this technique is progressing slowly. To address this, we summarize the indications for cryotherapy for rectal cancer based on the literature and clinical summaries to provide a reference for our peers.

## 2 Factors affecting cryotherapy

### 2.1 Freezing temperature and the size of ice balls

To achieve cell necrosis, cryotherapy usually requires a temperature below –40°C (5). However, tumor cells *in vivo* are typically destroyed when the temperature drops below –25°C (6). The core temperature of ice balls used in cryotherapy is typically below –25°C, while the temperature at the perimeter is typically around 0°C (7). It has been reported that the temperature just inside the ice ball, within 5 mm of the perimeter, can reach –20°C to –50°C; this means that the ice ball needs to extend at least 5 mm beyond the tumor boundary (8). Typically, two freeze–thaw cycles, which can greatly increase the effect of ablation, are required (9). The absence of vital organs or tissues within 5 mm of the tumor is a prerequisite for avoiding serious complications.

### 2.2 Tumor site

The length of the rectum is typically 12–15 cm. Because of a lack of mesenteric protection, cryotherapy in the rectum can lead to serious complications, such as perforation in the upper two-thirds of the rectum. The low rectum has a length of around 7 cm (10), and the distance from the peritoneal reflexes to the anus varies between individuals. The rectum below the peritoneal reflexes is protected by the perirectal mesangium, which makes this part of the rectum suitable for cryoablation. Cryotherapy cannot be recommended for the rest of the rectum because of the lack of this protection. In the case of tumors in the anterior wall of the rectum, cryotherapy is associated with a high risk of complications, such as rectovaginal fistula or rectourethral fistula, because of the proximity of the vagina or urethra, so physicians need to achieve an accurate ice ball size. Operators can identify the tumor characteristics before the procedure using pelvic CT or magnetic resonance imaging (11).

### 2.3 Tumor histology

It is important to perform a biopsy to characterize the tumor before cryotherapy, identifying pathological types such as poorly differentiated adenocarcinoma, mucinous adenocarcinoma, signet ring cell carcinoma, and undifferentiated carcinoma; these types of tumor are highly invasive and may recur locally after freezing, so cryotherapy is not recommended. The main histological types suitable for cryotherapy are moderate- or high-grade adenocarcinoma, high-grade intraepithelial neoplasia, villous tubular adenoma, and squamous cell carcinoma. Patients with highly invasive tumors who refuse abdominoperineal combined resection may receive cryotherapy as a palliative treatment. By prolonging the freezing time, increasing the number of freeze–thaw cycles, or adopting the strategies of fast freezing and slow dissolution, the degree of necrosis of tumor cells can be increased (12); however, the consequent complications are worthy of attention.

### 2.4 Tumor size

To ablate the tumor completely, the diameter of the ice ball needs to be increased as the tumor size increases. When the necrotic volume caused by the ice ball increases, the risk of complications, such as bleeding, infection, and rectal stenosis, increases accordingly. Gu and Lu (13, 14) report that half of the circumference is the threshold between radical freezing and palliative freezing according to the precept formulated by the second national seminar on cryotherapy of anorectal cancer (held from November 15–19, 1984 in Fuzhou, China). Therefore, cryoablation typically targets tumors occupying one-third of the circumference.

### 2.5 Tumor stage

In low rectal cancer, the T stage reflects the extent of a tumor’s invasion of the intestinal walls. The low rectum is an extraperitoneal organ without visceral peritoneum; the T4 stage can be defined as invasion of adjacent organs or pelvic walls (15). The extent of freezing should also increase with the extent of tumor infiltration from the mucosal layers to the outside of the intestinal walls. However, in T4 tumors the periphery cannot be completely destroyed by cryotherapy, and such tumors are more likely to recur. Theoretically, the vast majority of T3 tumors without lymph node metastasis, as determined by preoperative MRI evaluation, can still receive radical cryoablation.

### 2.6 Lymph node metastasis

Rectal and anal cancers metastasize mainly to the lymph nodes. Cryoablation is a type of local ablation that principally targets the primary tumor. In most cases, the ice ball cannot cover the lymph nodes completely, resulting in insufficient cold ablation of the lymph nodes, meaning that, once lymph node metastasis has occurred, the risk of tumor recurrence rate is significantly increased. Even with peri-intestinal lymph node metastasis, it is still impossible to evaluate whether or not there are small lymph node metastases in D2 lymphadenectomy because of the lack of total mesorectal excision. In addition, intraoperative monitoring is unable to evaluate the status of lymph node ablation. It is helpful for us to identify the presence of lymph node metastasis though preoperative MRI examination (11, 16).

### 2.7 Mesorectal fascia status, extramural vascular invasion, vascular lymphatic vessel infiltration, and perineural infiltration

If these four indicators are positive, this indicates that the tumor has broken through the rectal fascia and may have reached the extramural vessels or invaded the nerves or vascular lymphatic vessels. As cryotherapy does not allow the microscopic reaction to be evaluated imaging, it is not recommended in such cases. MRI can help prolong patients’ overall survival by facilitating evaluation of the status of the four indicators above.

### 2.8 Sex

There are anatomical differences between men and women. In men, the anterior rectum is adjacent to the urethra, whereas in women it is adjacent to the vagina. When tumors located in the anterior wall of the rectum are targeted by cryotherapy, the ice ball can include, in men, some of the prostate and urethra and, in women, some of the rectovaginal diaphragm and vaginal wall, which can result in damage to the urethra or vaginal wall, respectively, and may lead to rectourethral fistulas or rectovaginal fistulas, respectively. In the case of tumors in the anterior rectal wall, the closer the tumor is to invading the urethra or vaginal wall, the greater the risk of rectourethral fistulas or rectovaginal fistulas. It is therefore crucial to determine the appropriate size of the ice ball. It is especially important to avoid creating fistulas when cryotherapy ablation is palliative.

## 3 Factors affecting safety

### 3.1 Patient comorbidities

Cryotherapy has many advantages, including the fact that it is quick and easy to administer and, as anesthesia is simple, it carries a low risk. However, it is important to be aware of certain comorbidities that are contraindications to cryotherapy. For example, cryotherapy in patients with hematological disorders associated with coagulation dysfunction, who may be taking anticoagulants orally during the perioperative period, may lead to bleeding or hemorrhagic shock. Some other conditions, such as pregnancy, uremia, and cardiopulmonary or liver dysfunction, are also contraindications.

### 3.2 Tumor-related complications

#### 3.2.1 Rectovaginal fistulas or rectourethral fistulas

If the tumor is located in the anterior rectal wall, cryotherapy is not recommended in patients with complications such as rectovaginal fistulas or rectourethral fistulas because cell necrosis will lead to enlargement of the fistula.

#### 3.2.2 Pelvic or peritumoral infection

Tumors may rupture or perforate as they progress, which may lead to infection. Because cryoablation can lead to edema and necrosis, the infection may be aggravated by cryoablation. In such cases, patients should be treated with cryotherapy cautiously as a palliative treatment.

#### 3.2.3 Cachexia

Patients with cachexia should not be treated with cryotherapy, as the tumor is not the primary concern and cryoablation may cause “cold shock” or accelerate the development of cachexia.

## 4 Cryotherapy or local resection

The indications for cryotherapy can be broadened on the basis of local resection. The ice ball can be much larger than the range of a local resection. Similarly, the remission rate of tumors is much higher in those treated with cryotherapy than in those treated with local resection. At the same time, the destruction of tissue by cryoablation goes far beyond the scope of the tumor. We can monitor the extent of ablation, using ultrasonography or CT, to ensure its effectiveness.

## 5 Indications and contraindications for cryotherapy

With developments in imaging, histology, and cytology, we can diagnose tumors, evaluate their characteristics and determine T stage more accurately. There are two main categories of cryosurgery: radical cryoablation and palliative cryoablation.

### 5.1 Radical cryotherapy

#### 5.1.1 The concept of radical cryoablation

All tumors are ablated using cryoablation. In long-term follow-up, no tumor recurrence, lymph node metastasis, or organ metastasis have been reported (13, 14).

#### 5.1.2 Benefits of radical cryoablation

The benefits of radical cryoablation are as follows: (1) complete tumor elimination, with no recurrence or metastasis reported in long-term follow-up; (2) greater likelihood of organ (e.g., anus) preservation; (3) avoidance of low anterior proctectomy syndrome; (4) low risk of anesthesia or surgery; and, (5) greater quality of life.

#### 5.1.3 Indications for radical cryoablation

Radical cryotherapy should not be undertaken in patients who have a strong desire to preserve their anus or if preoperative assessment suggests that the patient is medically unfit for the procedure. Considering the safety or efficacy of the technique were mentioned in front matter, indications for radical cryotherapy include the following: (1) solitary neoplasm; (2) a distance from the top of the tumor to the anus of < 7 cm; (3) appropriate tumor size (if the tumor occupies one-third or less of the intestinal cavity circumference, cryoablation is likely to be appropriate); (4) moderate- or high-grade adenocarcinoma, high-grade intraepithelial neoplasia, villous tubular adenoma, or squamous cell carcinoma; and, (5) clinical stage cT0–2N0M0.

### 5.2 Palliative cryotherapy

#### 5.2.1 Definition of palliative cryotherapy

Palliative cryotherapy means partial ablation of the tumor, which is likely to recur or metastasize.

#### 5.2.2 Benefits of palliative cryoablation

Patients may gain the following benefits from palliative cryoablation: (1) reduced tumor burden; (2) partial or complete relief from symptoms; (3) the availability of a treatment despite inoperability; and, when cryoablation is combined with chemotherapy, (4) reduction in the size of distant metastases due to the “distal effect” (17).

#### 5.2.3 Application of palliative cryotherapy

Palliative cryotherapy is appropriate for patients with inoperable tumors or who wish to preserve the anus, i.e., who have refused abdominoperineal combined resection. It is also applicable if multiple comorbidities make radical cryoablation infeasible. The main indications for palliative cryotherapy include the following: (1) the distance from the top of the tumor to the anus is < 7 cm; (2) the clinical stage is cT0–2N0M0, with mesorectal fascia/extramural vascular invasion/vascular lymphatic vessel infiltration/perineural infiltration, or the disease is too advanced to be effectively treated with radical cryotherapy.

#### 5.2.4 Cryotherapy after neoadjuvant therapy

According to the Chinese colorectal diagnosis and treatment guidelines (11), in principle, T3 tumors should be treated with neoadjuvant chemotherapy before radical cryosurgery. In this case, doctors should proceed with caution. It is difficult for tissues to heal after neoadjuvant radiotherapy because of the occlusion of blood vessels and tissue edema After cryotherapy it is more difficult for ulcers to heal; therefore, recovery time can be significantly prolonged (18, 19). After neoadjuvant radiotherapy and chemotherapy, it is necessary to re-evaluate the characteristics of the tumor and the feasibility of cryotherapy.

### 5.3 Contraindications to cryoablation

#### 5.3.1 Relative contraindications

Considering the treatment efficacy of cryoablation and safety factors, relative contraindications include the following: (1) familial adenomatous polyposis in low rectal cancer or anorectal cancer (familial adenomatous polyposis is associated with high rates of recurrence and may recur after cryoablation); (2) radiotherapy for rectal cancer in the preceding 6–8 weeks (increased recovery time); (3) rectovaginal fistula or rectourethral fistula (there is a high risk of expanding the fistula after cryotherapy); (4) hypoimmunity (increased risk of perirectal infection after cryoablation); and, (5) uremia (increased bleeding risks).

#### 5.3.2 Absolute contraindications

To avoid potentially uncontrollable complications, absolute contraindications to cryoablation are as follows: (1) serious blood system disease with coagulation dysfunction; (2) long-term oral anticoagulant treatment; (3) vascular embolic disease in the preceding 3 months; (4) pregnancy; (5) cachexia; and, (6) an American Society of Anesthesiology (ASA) physical condition and surgical risk score of IV or V.

## 6 Summary and outlook

It is very important to evaluate the patient’s condition before treatment and to select an appropriate plan according to the patient’s needs. Cryoablation has a unique advantage in organ preservation. By applying the technique only to patients with the appropriate indications, the use of cryotherapy can improve quality of life and long-term prognosis among those who undergo the procedure. Meanwhile, cryoablation provides an effective method for those who cannot undergoing surgery. It is an excellent complementary treatment for rectal cancer and anorectal cancer. It is of great significance for cryosurgery, and has established indications. We have summarized the indications and contraindications for cryosurgery to facilitate clinical application and promote appropriate treatment. However, these indications should keep pace with developments in medical technologies to ensure that patients benefit as much as possible from cryotherapy.

## Author contributions

XJ: conceptualization, methodology, software, data curation, and writing (original draft preparation). ZJ: visualization and investigation. XL: software and validation. YH: revising. FY: writing (reviewing and editing). All authors contributed to the article and approved the submitted version.

## References

[1] Liu Z Li Z Zhang Y Zhou T Zhang J You W. Interpretation of 2020 global cancer statistical report. Electron J Compr Tumor Ther (2021) 7(02):1–14. doi: 10.12151/JMCM.2021.02-01

[2] Lu C Luo X Wang W Gao W Li Yu C. Rational selection and application of different surgical methods in low rectal cancer. Chin J Endosc Surg (Electron Ed) (2020) 13(02):124–8. doi: 10.1186/s12880-021-00706-0

[3] Néron S Perez S Benc R Bellman A Rosberger Z Vuong T. The experience of pain and anxiety in rectal cancer patients during high-dose-rate brachytherapy. Curr Oncol (2014) 21(1):e89–95. doi: 10.3747/co.21.1741 PMC3921053 24523626

[4] Tekkis P Tait D Cunningham D Brown G. Is organ preservation in rectal cancer ready for prime time? Lancet (2018) 391(10139):2480–2. doi: 10.1016/S0140-6736(18)31324-2 29976455

[5] Yakkala C Denys A Kandalaft L Duran R. Cryoablation and immunotherapy of cancer. Curr Opin Biotechnol (2020) 65:60–4. doi: 10.1016/j.copbio.2020.01.006 32088576

[6] Clarke DM Robilotto AT Rhee E VanBuskirk RG Baust JG Gage AA. Cryoablation of renal cancer: variables involved in freezing-induced cell death. Technol Cancer Res Treat (2007) 6(2):69–79. doi: 10.1177/153303460700600203 17375969

[7] Saliken JC McKinnon JG Gray R. CT for monitoring cryotherapy. AJR Am J Roentgenol (1996) 166(4):853–5. doi: 10.2214/ajr.166.4.8610562 8610562

[8] Ismail M Nielsen TK Lagerveld B Garnon J Breen D King A. Renal cryoablation: Multidisciplinary, collaborative and perspective approach. Cryobiology (2018) 83:90–4. doi: 10.1016/j.cryobiol.2018.06.002 29890126

[9] Hoffmann NE Bischof JC. The cryobiology of cryosurgical injury. Urology (2002) 60(2 Suppl 1):40–9. doi: 10.1016/S0090-4295(02)01683-7 12206847

[10] Qiu X Lin G. Research progress of laparoscopic anus preserving surgery for low rectal cancer. Chin J Endosc Surg (Electron Ed) (2021) 14(02):65–9.

[11] Gu J Wang J Shen L Xu R Li J Zhang Z. Chinese Standard for diagnosis and treatment of colorectal cancer (2020). Chin J Pract Surg (2020) 40(06):601–25. doi: 10.19538/j.cjps.issn1005-2208.2020.06.01

[12] Erinjeri JP Clark TW. Cryoablation: mechanism of action and devices. J Vasc Interv Radiol (2010) 21(8 Suppl):S187–91. doi: 10.1016/j.jvir.2009.12.403 PMC6661161 20656228

[13] Gu B. Application of cryosurgery in anorectal surgery. Gen Surg clinic (1996) 03):149–51.

[14] Lu Z. Clinical experience of cryotherapy for 25 cases of low rectal cancer. Chin Community Physician (Med Spec) (2010) 12(09):38. doi: 10.3969/j.ssn.1007-614x.2010.09.035

[15] Gu J Zhang X. Evaluation of resectability of low rectal cancer. Chin J Gen Surg (Electron Ed) (2015) 9(03):161–5. doi: 10.3877/cma.j.issn.1674-3946.2015.03.051

[16] Zhou Y Yang R Wang Y Zhou M Zhou X Xing J. Histogram analysis of diffusion-weighted magnetic resonance imaging as a biomarker to predict LNM in T3 stage rectal carcinoma. BMC Med Imaging (2021) 21(1):176. doi: 10.1186/s12880-021-00706-0 34809615 PMC8609786

[17] Yuan F Zhou W Zhang J Zhang Z Zou C Huang L. Anticancer drugs are synergistic with freezing in induction of apoptosis in HCC cells. Cryobiology (2008) 57(1):60–5. doi: 10.1016/j.cryobiol.2008.06.001 18586021

[18] Hu K Xiao Y. Advantages and disadvantages of neoadjuvant short - range radiotherapy and long - range radiotherapy for rectal cancer. Chin J Gastrointest Surg (2017) 20(07):773. doi: 10.3760/cma.j.issn.1671-0274.2017.07.013

[19] Li X Li Q. Controversy and progress of adjuvant chemotherapy after neoadjuvant radiotherapy and chemotherapy for locally advanced rectal cancer. Chin J Gastrointest Surg (2019) 06):594–6. doi: 10.3760/cma.j.issn.1671?0274.2019.06.015 31238639

